# Therapeutic Effects of Oleuropein in Improving Seizure, Oxidative Stress and Cognitive Disorder in Pentylenetetrazole Kindling Model of Epilepsy in Mice

**DOI:** 10.22037/ijpr.2019.14212.12209

**Published:** 2020

**Authors:** Samira Asgharzade, Zahra Rabiei, Sana Rabiei, Elham Bijad, Mahmoud Rafieian-Kopaei

**Affiliations:** a *Cellular and Molecular Research Center, Basic Health Sciences Institute, Shahrekord University of Medical Sciences, Shahrekord, Iran. *; b *Medical Plants Research Center, Basic Health Sciences Institute, Shahrekord University of Medical Sciences, Shahrekord Iran. *; c *Department of Seafood Processing, Faculty of Marine Sciences, Tarbiat Modares University, Noor, Iran.*

**Keywords:** Oleuropein, Seizure, Pentylenetetrazol, Anti-inflammatory, Antioxidant

## Abstract

Prolonged epileptic seizures are the cause of neuronal death and brain damage. Lesions in different regions of the brain can lead to memory loss and cognitive disorders. It is therefore essential to seek out new neuroprotective drugs. Our aim was to investigate the therapeutic effects of oleuropein in improving seizure, oxidative stress, and cognitive disorder in pentylenetetrazole (PTZ) kindling model of epilepsy in mice. Mice were randomized to four groups; negative control group intraperitoneally receiving PTZ for 10 days, oleuropein group receiving oleuropein (20 mg/kg) 30 min before PTZ administration, positive control group receiving diazepam 30 min before PTZ administration and flumazenil group receiving flumazenil and then oleuropein 30 min before PTZ administration. Epilepsy severity was investigated after final administration of PTZ. Then hippocampal tissues were removed and stored at -70 °C until measurements of the interleukin-1 (IL-1) and glutamate transporter 1 (GLT-1) gene expression were conducted. Oleuropein treatment caused a significant increase in seizure latency and a significant decrease in total frequencies of head ticks, head and upper limbs seizures, the whole body seizures, frequent spinning and jumping and tonic seizures in PTZ receiving mice. IL-1 expression decreased in oleuropein group and GLT-1 levels did not change significantly in this group. Oleuropein treatment caused significant improvement of passive avoidance memory in PTZ receiving mice in shuttle box. Oleuropein can decrease PTZ-induced seizures and memory disorders due to its antioxidant and anti-inflammatory properties and is thus recommended to be used for production of anti-epileptic drugs.

## Introduction

Epilepsy is the second leading neurological disorder following stroke and is characterized by unpredictable and periodical seizures. Seizure refers to a transient behavioral change due to the rhythmic and synchronized discharges of a set of neurons in the central nervous system (1). In epilepsy, a wide spectrum of pathological changes in neurons and axonal sprouting, inflammation and gliosis occur during the seizure-free latent period following epilepto-genesis and may last for several years (2). Prolonged epileptic seizures induce neuronal death in several subfields branches of the hippocampus, amygdala, thalamus, cerebellum, piriform, and the entorhinal cortex in both humans and animals (3). Neuroprotection is one of the promising strategies to prevent and improve pathological outcomes in epilepsy. However, protection against neuronal damage in the hippocampus alone is not sufficient to prevent epilepsy. Neuronal damage is important in the brain damage-associated behavioral, learning and memory disorders following epilepsy as well as reducing resistance to anti-epileptic drugs (AEDs) (3). Excessive expression of cytokines in astrocytes reduces seizure threshold and the frequency of spontaneous seizures (4). A rapid inflammatory response begins in glial cells following seizures due to chemoconvulsantsor electrical stimulation (5). Through inflammatory response, certain cytokines such as interleukin-1β (IL-1β), interleukin-6 (IL-6) and tumor necrosis factor-alpha (TNF-α) initiate downstream inflammatory cascade in the nerve and epithelial cells of the brain-blood barrier (BBB) (including activation of COX-2, NF-kB, complement system, chemokine, and acute-phase proteins) (5).

Therefore, an efficient step to treating epilepsy can be studying the neuroprotective and anti-inflammatory drugs. Oleaeuropaea is one of the most well-known Asian herbs and has been in the diet of people for more than 2000 years (6). The neuroprotective and anti-inflammatory properties of Oleaeuropaea, have been demonstrated (7). As the main bioactive compound of Oleaeuropaea, oleuropein is found in abundance in the unprocessed fruit and leaf of this plant (6). Oleuropein has been found to exert various neuroprotective effects in different neurological disorders including hippocampal neuronal damage in cerebral ischemia (8), brain damage following hypoxia-reoxygenation in rat model of type 1 diabetes (9), high-fat diet-induced audiogenic seizure (10), rat model of spinal cord damage (11), and neurotoxicity (12). Oleuropein neuroprotective property is due to free radical inhibitory, antioxidant, lipid peroxidation inhibitory, anti-inflammatory, glutathione restoring, and anti-apoptotic properties (13). This study was aimed at investigating the phytohemical and neuroprotective effects of oleuropein in the mice with pentylenetetrazole (PTZ)-induced epilepsy as well as the anti-inflammatory and antioxidant effects of oleuropein in epilepsy. 

## Experimental

*Drugs and active compounds*

**Figure 1:**
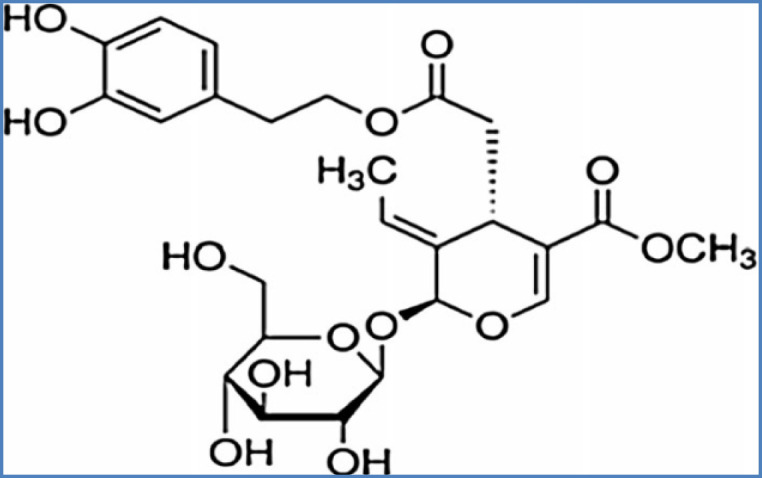
Oleuropein chemical structure

Diazepam, PTZ, oleuropein (Figure 1) and flumazenil were purchased from Sigma (St. Louis, MO, USA). Oleuropein and PTZ were solid and were dissolved in normal saline. 

*Biochemical tests*

*Measuring oleuropein antioxidant effect*

Oleuropein antioxidant effect was measured by the 2, 2-diphenyl-1-picryl Hydrazyl (DPPH) assay and the trolox equivalent antioxidant capacity (TEAC) assay. 

*The DPPH assay*

Oleuropein (3.5, 6.25, 12.5, 25, 50 and 100 µg/mL) was first prepared and equal amount of the DPPH solution (1 mg/mL) was added to oleuropein at all concentrations. The resulting solution was kept in the dark at room temperature for 15 min, the absorbance values were measured at 517 nm using a spectrophotometer and then the activity of the DPPH radical inhibition was calculated (14). 

IC50 (%) = (Acontrol-Asample)/Acontrol×100 

IC50 is the concentration of the solution in which 50% of the DPPH radical was scavenged

*The TEAC assay*

To prepare azino-bis 3-ethylbenzothiazoline-6-sulfonic acid (ABTS), an aqueous solution of ABTS (7 mM) was prepared. Potassium persulfate was added to this ABTS solution to a final concentration of 2.45 mM and the resulting solution was left in the dark at room temperature for 16 hours. Meanwhile, ABTS was converted to its radical cation by addition of potassium persulfate. Then, oleuropein at 75, 125, 250, and 500 µg/mL was prepared and 20 µg/mL of each concentration of the sample mixed with 2mL of ABTS^•+ ^and the absorbance was read at 734nm. The results were expressed as TEAC value (the ability to inhibit ABTS radical by the Trolox standard) (15).

*Metal chelating assay*

Briefly, oleuropein (30, 50 and 100 mg/mL) was mixed with FeCl2 (0.5 mM, 2 mM) and ferrozine (0.2 mL, 5 mM) and shaken. After 10 min, the absorbance was read using a spectrophotometer at 562nm. EDTA was used to plot the standard curve. The percentage of the ferrous ion-chelating capacity was measured by the equation below:

(Absorbance of control - Ab of sample)/Ab of control x 100 (16).

*Reducing power assay*

Reducing power of a compound represents its electron-donating ability. Oleuropein (1 mM) at 25, 50, 100, 200, and 300 µg/mL was mixed with phosphate buffer (0.2 M, pH = 6.6) and 1% potassium ferricyanide (K_3_Fe (CN)_6_) and the resulting solution was left to incubate at 50 °C for 2 min. Chloro-acetic acid was added to stop the reaction. The mixture was centrifuged at 3000 rpm for 10 min, the supernatant was mixed with H_2_O_2_ and ferric chloride 1% and the absorbance was measured at 700 nm (17).

*Hydroxyl radical scavenging assay*

First, 1,10-phenanthroline (1 mL, 1.865 mM) was mixed with 2 mL of oleuropein (25, 50, and 100 µg/mL), and FeSO4 (1 mL, 1.865 mM) then added to the resulting mixture. The reaction was started by the addition of H_2_O_2_ (0.03%). The resulting solution was incubated in water bath at 37 °C for 60 min and the absorbance was read at 536nm. The scavenging activity of hydroxyl radical was measured by the equation below: 

HRSA (%) = [(Absorbance of sample_Ab of A negative control)/ (Ab of blank _ Ab of negative control)] ×100 (18).

*Animals*

The mice weighing 25-30g were purchased from the Pasteur Institute of Iran (Tehran, Iran) and housed under (21 ± 3) °C and 12 h light/12 h cycles as they had free access to water and standard food. All tests and manipulations were conducted according to the guidelines of Institutional Animal Care and the Medical Ethics Committee (Medical Plants Research Center and Institute of Basic Sciences Research) of Shahrekord University of Medical Sciences).

*Behavioral tests*

*Grouping of mice*

The mice were assigned to four groups of 10 each as follows:

PTZ (negative control) group: Intraperit-oneally administered with PTZ (35 mg/kg) once every 48 hours for 10 days;

Oleuropein group: Receiving PTZ once every 48 h and intraperitoneally administered with 20 mg/kg of oleuropein daily 30 min before PTZ injection, for 10 days;

Diazepam group: Receiving PTZ once every 48 h for 10 days and intraperitoneally administered with diazepam (2 mg/kg) 30 min before PTZ injection on day 10; and 

Flumazenil group: Receiving PTZ for 10 days and flumazenil (2 mg/kg) 10 min before intraperitoneal administration of PTZ (20 mg/kg) on day 10. PTZ was administered 30 min after the Oleuropein injection. 

*The model of PTZ-induced epilepsy*

PTZ is used to induce myoclonic seizure in biochemical and pharmacological studies (14). In the current study, PTZ was intraperitoneally administered at 35 mg/kg every 48 h until day 10 and at 60 mg/kg, the lethal dose of PTZ, on day 10. Other drugs were intraperitoneally administered 30 min before PTZ injection. After injection of PTZ at lethal dose, the mice were observed for head ticks, head seizure, and upper limb sudden jerk, whole body seizure and standing on hind legs, tonic seizures and frequent spinning and jumping for 30 min. Then, the mice either died or returned to normal condition (19). The rats were not required to pass through all steps of interest and were likely to pass through certain steps so quickly that they could not be noticed. 

*Passive avoidance test*

Passive avoidance test was conducted by a passive avoidance apparatus. This apparatus consists of a bright chamber, connected to dark chamber (the floor of the dark chamber is a metal grid) and a moving blade between the two chambers. This test was conducted on each mouse during four consecutive days. 

On the first two days of the test, the mice were individually allowed to explore freely for 5 min in the apparatus to acclimate to it, and on the third day, an acquisition test was conducted. The mice were then left in the bright chamber and, 2 min later, the guillotine door was opened and the initial latency to enter the dark chamber was recorded. 

In the dark chamber, an electrical shock (1 mA/sec) was exerted to the mouse so that they only paddled, in this test; the initial latency to enter the dark chamber that was recorded. Twenty four hours later, the mice were individually placed in the bright chamber to continue the test. Twenty four hours later, the rat was placed in the bright room, but without the foot-shock, and the interval between being left in the dark chamber and entering the bright one was measured and considered to be the secondary latency (up to 60 sec) (20).

*Measuring membrane lipid peroxidation in serum*

Malondialdehyde (MDA) levels in the hippocampal tissues were measured using the thiobarbituric acid reactive substance (TBARS). MDA is one of the end products of lipid peroxidation of polyunsaturated fatty acids and is used as a lipid peroxidation index (21). Plasma (100 μL) was mixed with 1.8% sodium dodecyl sulfate (100μL) and thiobarbituric acid (2.5 mL) and then heated in boiling water bath (at 95 °C) for 60 min. The reaction was stopped by placing the tube on ice. After a 10 min centrifugation at 4000 rpm, the absorbance of the supernatant was read at 535 nm using a spectrophotometer. 

*Determining the ferric reducing antioxidant power (FRAP) of serum and hippocampal tissue*

The FRAP assay, one of the most common methods to determine total antioxidant activity, was conducted according to Strain and Benzie protocol (21). This method is based on the tissue fluid’s ability to reduce ferric ions (Fe^3+^) to ferrous ions (Fe^2+^) in the presence of TPTZ (tripyridyl-s-triazine). The reducing power of the tissue fluids is spectrophotometrically measured by increase in blue-colored TPTZ-Fe^2+^ complex. Briefly, 300 mM/L acetate (pH 3.6), 10mM/l TPTZ, and 20 mM/L FeCl3 in a ratio of 10:1:1 ratio was mixed to give FRAP reagent. Then, the homogenates of the serum or tissue samples were mixed with the FRAP reagent and then left in 37 °C water bath for 10 min. The absorbance at 593nm was recorded and compared with the standard curve plotted by the standard FeCl3.6H2O solution and the values were expressed in mL/gkw (22). 

*Molecular tests*

*RNA extraction and cDNA synthesis, real-time PCR*

Hippocampal tissues were removed from the brains and stored at -70 °C. RNA extraction was conducted using Trizol (Invitrogen, Carlsbad, CA, USA) according to the manufacturer’s instructions. For reverse transcription, 3000ng of the total RNA of each sample was used. Real-time PCR was conducted using a cDNA synthesis kit (Thermo Science) according to the manufacturer’s instructions. Specific primers were designed by the Oligo ver. 6.7.1.0 (National Biosciences Inc.). Table 1 shows the sequence of the target genes and specific primers. 

Quantification of the mRNA levels of IL-1β and GLT-1 genes was conducted by Rotor-Gene 3000 (Corbett, Australia). The reaction was conducted with a final volume of 13 μL in a 0.1 μL microtube. The compounds of each reaction consisted of 1 μL of the synthesized cDNA, 5μL of SYBR Premix Ex Taq (TliRNase H Plus) (Takara, Japan), and 0.2 μL of each of the forward and reverse primers (10 pM). 

The temperature protocol consisted of denaturation at 95 °C for 10 min followed by 40 cycles of denaturation at 95 °C for 15 s, annealing at the specific temperature of the specific primers (Table 1) for 30 s, and extension at 72 °C for 30 s.

This protocol was conducted for each sample in duplicate. GADPH (glyceraldehyde-3-phosphate dehydrogenase) was used as housekeeping gene to normalize the expression of the target genes. The relative expression levels of the target cDNAs were quantified by the 2^ΔΔCts^ method by 2^ΔΔCts^ method. 

## Results

*Biochemical tests*

*Oleuropein antioxidant property*

*DPPH levels*

The results demonstrated that the anti-radical activity of oleuropein increased with increasing its concentration. In addition, the EC50 of oleuropein was derived 11.7 μg/mL. EC50 was directly correlated with oleuropein antioxidant activity.

*TEAC assay*

*Metal chelating assay*

The ferrous ion-chelating capacity of oleuropein increased with increasing its concentration. Ferrous ion-reducing power (%) of oleuropein at 50, 30, and 100 μg/mL was derived 17.75%, 21.07% and 22.20%, respectively. 

*Ferrous ion-reducing power*

The antioxidants with high ferrous ion-reducing power can exert potent effects in terminating damaging oxidative chain reactions. We observed that the ferrous ion-reducing power of oleuropein increased with increasing its concentration such that the absorbance was derived 0.007 at 25 μg/mL, with the highest absorbance (1.958) at 300 μg/mL and. The EC50 of oleuropein was derived 95.51 μg/mL. 

*Hydroxyl radical scavenging assay*

Hydroxyl scavenging activity (%) of oleur-opein at 50, 25 and 100 μg/mL was derived 4.98%, 4.88% and 8.8%, respectively. 

*Behavioral tests*

*Passive avoidance test*

**Figure 2:**
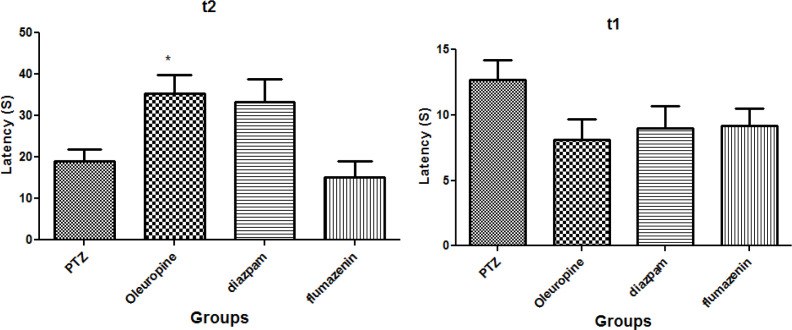
Oleuropein effect on mouse model of epilepsy; t_1_: Mean duration of initial latency; t_2_: Mean duration of secondary latency

^* ^Significant difference when compared to pentylenetetrazole group (*p *< 0.05).

Figure 2 illustrates the oleuropein effects on the initial and secondary latencies in the passive avoidance test. The results demonstrated that there was no significant difference in initial latency to enter the dark chamber among the groups (*p *< 0.05) (Figure 2 (t_1_)). Secondary latency in the oleuropein group significantly increased when compared to the PTZ group (*p *< 0.05) (Figure 2 (t_2_)), but secondary latencies in diazepam and flumazenil groups were not significantly different compared to the PTZ group.

*Seizure latency*

**Figure 3:**
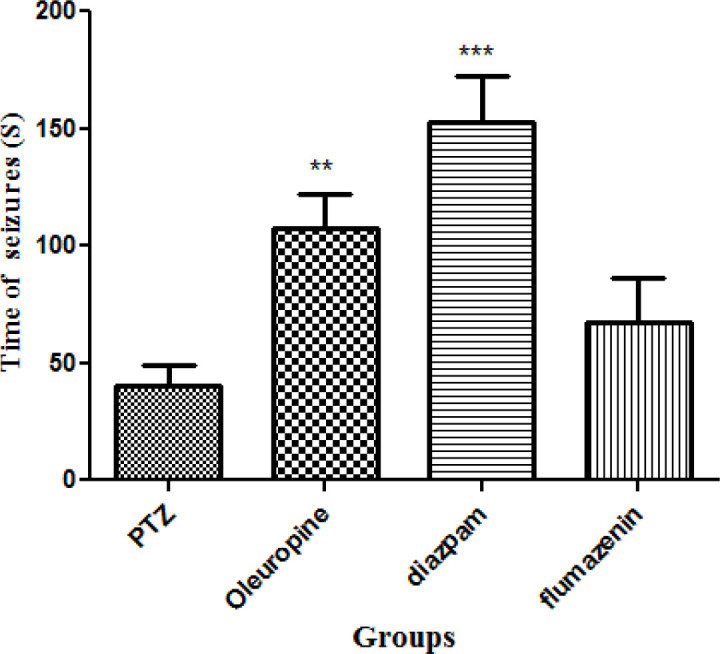
Seizure latencies in different groups of mice

^** ^Significant difference when compared to pentylenetetrazole group (*p *< 0.001);

^** ^Significant difference when compared to oleuropein group (*p *< 0.05).

Oleuropein caused significant increase in seizure latency in the PTZ receiving mice (*p *< 0.05). Seizure latency in the diazepam group was significantly higher when compared to the PTZ group (*p *< 0.001). Seizure latency was not significantly different between the flumazenil group and the PTZ group (Figure 3). 

*Total frequencies of head ticks*

**Figure 4:**
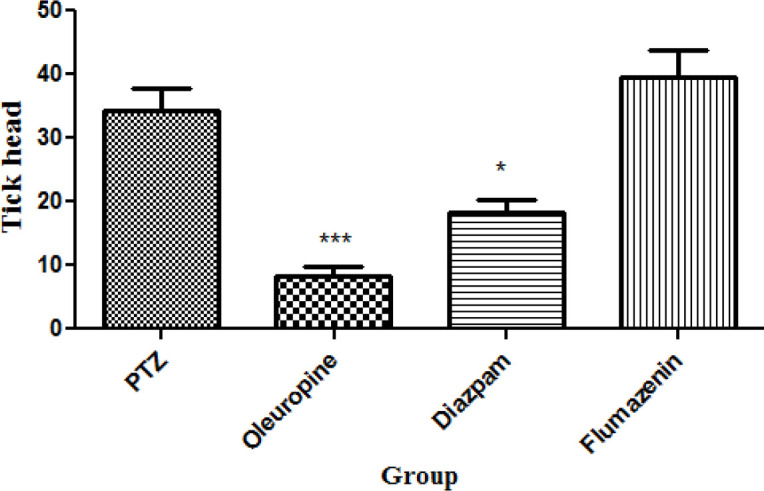
Total frequencies of head ticks in different groups of mice

^* ^Significant difference when compared to pentylenetetrazole group (*p *< 0.05 and 0.001, respectively)

Oleuropeinand diazepam treatment caused significant decrease in total frequencies of head ticks compared to PTZ treatment (*p* < 0.05 and 0.001, respectively) (Figure 4). 

*Total frequencies of head and upper limbs seizures*

**Figure 5:**
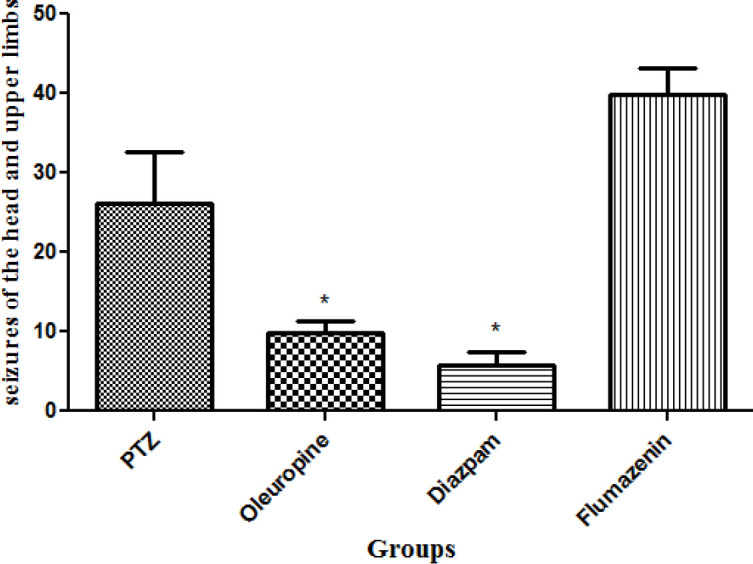
Total frequencies of head and upper limbs seizures in different groups of mice

^* ^Significant difference when compared to pentylenetetrazole group (*p*< 0.05).

Total frequencies of head and upper limbs seizures in the oleuropein group decreased significantly when compared to the PTZ group (*p* < 0.05) and the diazepam group (*p* < 0.05) (Figure 5). 

*Total frequencies of the whole body seizures*

**Figure 6.**
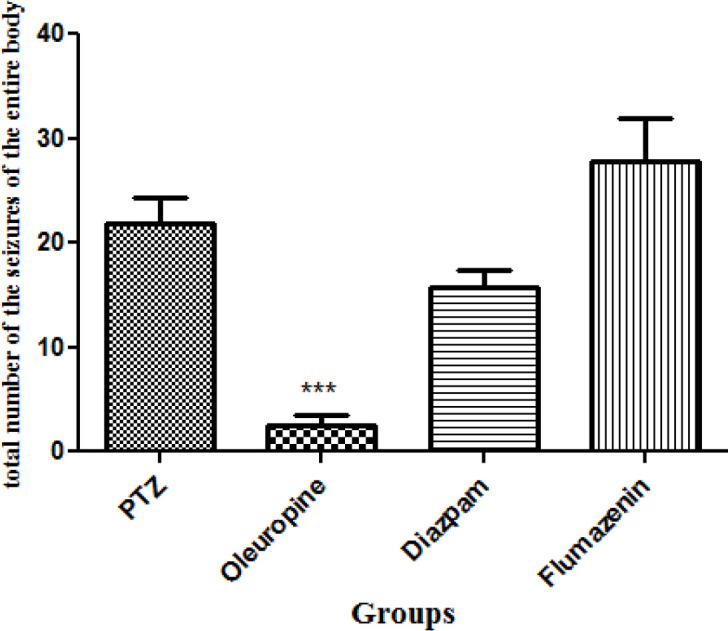
Total frequencies of the whole body seizures in different groups of mice

^*** ^Significant difference when compared to pentylenetetrazole group (*p* < 0.001)

Oleuropein treatment caused significant decrease in total frequencies of the whole body seizures in the PTZ receiving mice (*p *< 0.001). Total frequencies of the whole body seizures in the oleuropein group decreased significantly when compared to the diazepam group (Figure 6).

*Total frequencies of tonic seizures*

**Figure 7.**
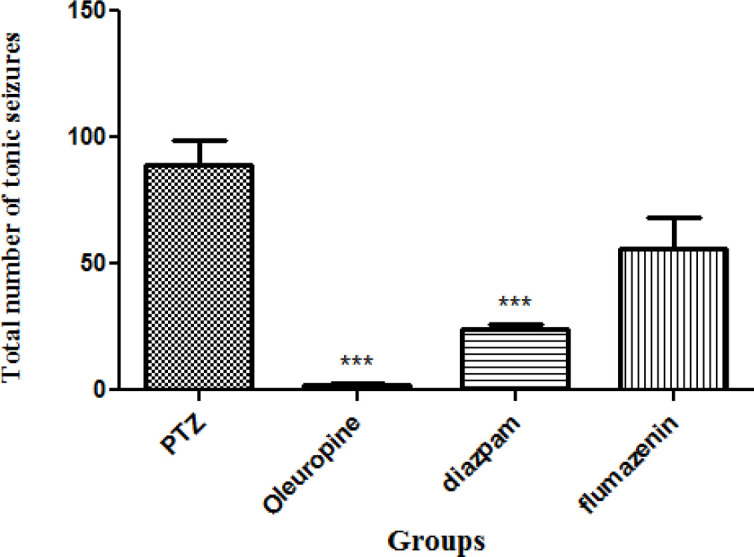
Total frequencies of tonic seizures in different groups of mice

^*** ^Significant difference when compared to pentylenetetrazole group (*p* < 0.001)

Tonic seizures in the oleuropein group and diazepam group decreased significantly when compared to the PTZ group (*p* < 0.001) (Figure 7). 

*Total frequencies of frequent spinning and jumping*

**Figure 8:**
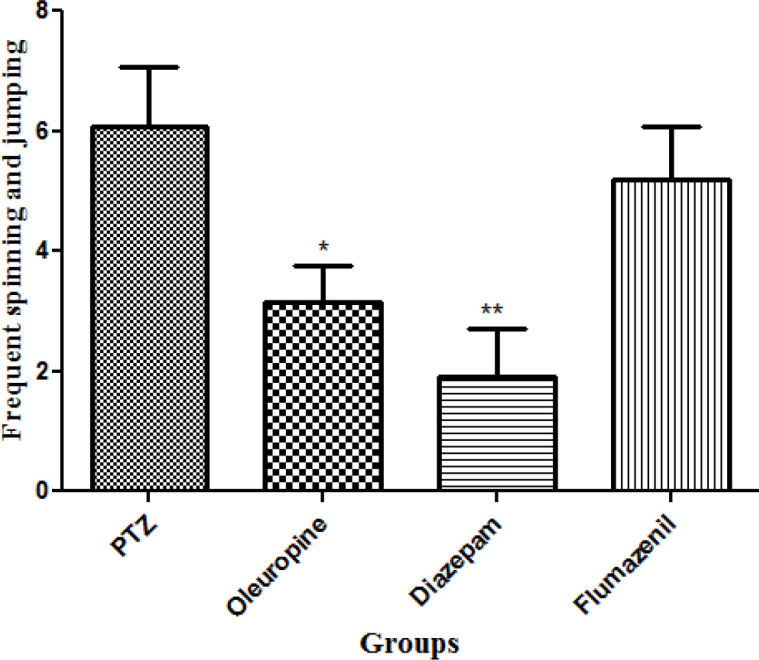
Total frequencies of frequent spinning and jumping in different groups of mice

^* ^Significant difference when compared to pentylenetetrazole group (*p* < 0.05);

^** ^Significant difference when compared to pentylenetetrazole group (*p* < 0.01)

Diazepam and oleuropein treatment caused significant decrease in total frequencies of frequent spinning and jumping in the PTZ receiving mice (*p* < 0.01 and 0.05, respectively) (Figure 8). 

*Membrane lipid peroxidation in serum*

**Figure 9:**
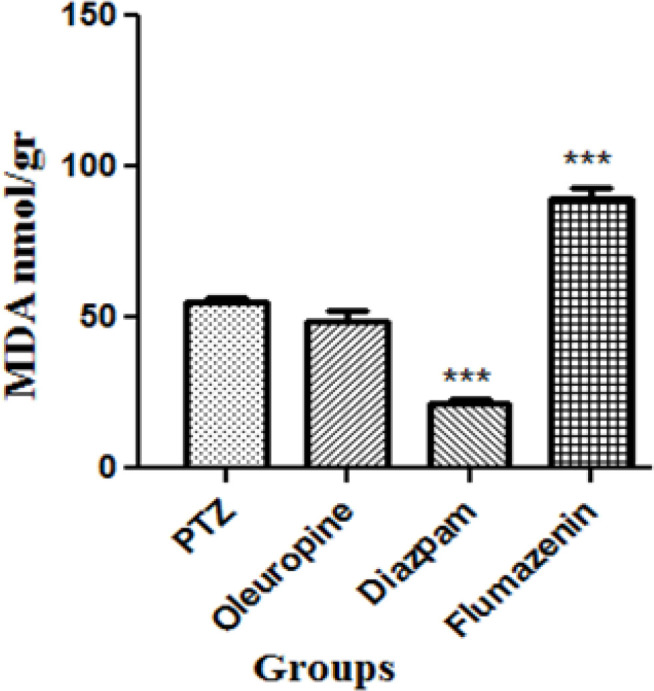
The malondialdehyde levels in brain in different groups of mice

^*** ^Significant difference when compared to pentylenetetrazole group (*p* < 0.001).

Serum MDA levels decreased significantly in the diazepam group and flumazenil group when compared to the PTZ group (*p* < 0.001), but did not change significantly in the oleuropein group when compared to the PTZ group (Figure 9). 

*FRAP of serum and hippocampal tissue*

**Figure 10:**
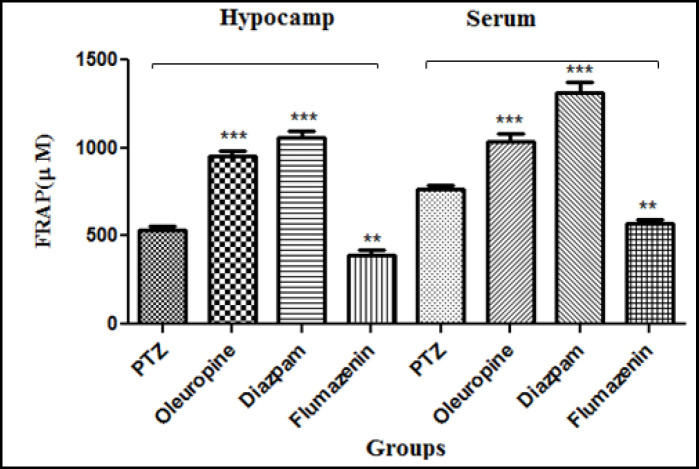
Ferric reducing antioxidant power of the serum and hippocampal tissues in different groups of mice

^*** ^Significant difference when compared to pentylenetetrazole group (*p* < 0.001);

^** ^Significant difference when compared to pentylenetetrazole group (*p* < 0.01).

Oleuropein caused increase in the antioxidant capacity of serum and hippocampal tissue through increasing the ferrous ion-reducing power. The FRAP of serum and hippocampal tissues increased significantly in the oleuropein group and diazepam group when compared to the PTZ group (*p* < 0.001), but did not change significantly in the flumazenil group when compared to the PTZ group (*p* < 0.01) (Figure 10). 

*Molecular tests*

*RNA extraction and cDNA synthesis, real-time PCR*

**Figure 11:**
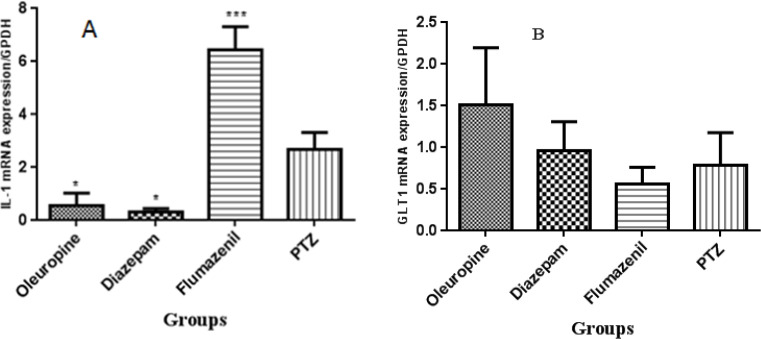
The interleukin-1β and glutamate transporter 1 genes expression in the hippocampal tissues in different groups of mice

^*** ^Significant difference when compared to pentylenetetrazole group (*p*<0.001);

^* ^Significant difference when compared to pentylenetetrazole group (*p*<0.05).

IL-1 gene expression decreased signifi-cantly in the oleuropein group and diazepam group when compared to the PTZ group (*p* < 0.05), but increased significantly in the flumazenil group when compared to the PTZ group (*p* < 0.001). GLT-1 gene expression did not change significantly in none of the groups (Figure 11). 

. 

**Table 1:** The sequence of primers

**Gene**	**Primer sequence**
GLT1	Forward:5- TCT GAC ACC ATC TCG TTC ACT-3 Reverse: 5-CTC TTA GTG TCC TTC TCG TTC CT-3
IL-1β	Forward:5-CCT CAC AAG CAG AGC ACA AG-3 Reverse:3-CAT TAG AA CAG TCC AGC CCA TAC-3
GPDH	Forward:5- GGTGGAGCCAAAAGGGTCAT-3 Reverse: 5- GGTTCACACCCATCACAAACAT-3

## Discussion

Given the wide prevalence of epilepsy and the low efficacy of synthetic drugs, it seems essential to seek out new herbal drugs with satisfactory efficacy and comparatively fewer side effects. We were therefore encouraged to investigate the therapeutic effects of oleuropein to improve seizure, oxidative stress, and cognitive disorders in PTZ kindling model of epilepsy in mice.

We did not observe any significant difference in initial latency to enter the dark chamber among the groups, but secondary latency in the oleuropein group significantly increased compared to the PTZ group. Oleuropein and diazepam caused significant increase in seizure latency and significant decrease in the total frequencies of head ticks, head and upper limbs seizures, frequent spinning and jumping, and tonic seizures in the PTZ receiving mice. Oleuropein also caused a significant decrease in the whole body seizures. PTZ leads to inhibition of GABA secretion by binding to picrotoxin site of the GABA receptor complex. Is inhibition the inhibitory neurotransmitter GABA and/or activation of n-methyl-o-aspartate receptor (NMDA) appear to be the trigger for inducing seizure due to PTZ (23). GABA receptor antagonists lead to development and progression of the seizure (24). However, GABA receptor agonists decrease the severity and duration of the seizures. Diazepam is a GABA receptor agonist, and it has been shown that it reduces the severity and symptoms of the seizure in animals and humans (13).

We did not observe any significant change in anticonvulsant effects in the oleuropein receiving mice after flumazenil (GABA receptor antagonist) treatment. Given no significant change in GLT-1 gene expression in the oleuropein group compared to the PTZ group, it is less likely for oleuropein to exert anticonvulsant effect through the GABAergic system.

Nitric oxide (NO) is a neurotransmitter and it released endogenously and participates in the excitatory transmission through NMDA receptors (25). Inconsistent results regarding the anticonvulsant and proconvulsant effects of NO in different models of seizure have been reported. 

For example, a study showed that NO production resulted in an end to NMDA (nonselective NOS inhibitor) induced seizure, and another study indicated that NO inhibition had an anticonvulsant effect on NMDA-induced seizure. Oleuropein increases the NO expression and production in the mouse macrophage. In addition, the anti-hypertensive effect of oleuropein through an increase in NO production has been reported (6, 25).

Rahimi et al. reported the antiepileptic effects of oleuropein through opioidergic/nitergic pathways and confirmed this report by blockade of opioid receptors and neuronal NO inhibitors (6). 

Oleuropein is a natural polyphenol belo-nging to the secoiridoids and has various pharmaceutical properties. Oleuropein has been reported to exert neuroprotective effects in different nerve damages including cerebral hypoxia-reoxygenation, audiogenic seizures related to a high-fat diet, hippocampal damage, cerebral ischemia, Alzheimer’s disease, and aging in different experimental models (12, 26). One of the most prominent features of this compound is its potent antioxidant activity, which is due to the presence of hydroxyl group in its chemical structure (1, 2-dihydroxybenzene moiety), preventing oxidation via donating hydrogen group (27). The free radical scavenging and metal chelating activities of oleuropein appear to be responsible for preventing membrane lipid peroxidation by metals. Oleuropein prevents the production of reactive oxygen species (anion superoxide, hydrogen peroxidase, and H_2_O_2_) and reactive nitrogen species [nitric oxide (NO), peroxynitrite and ONOO−]. Besides that, oleuropein was found to increase the levels of antioxidant enzymes such as superoxide dismutase, glutathione peroxidase, glutathione reductase, and catalase as well as non-oxidant enzymes such as glutathione, tocopherol, beta-carotene, and ascorbic acid in the diabetic rabbits and rats that fed a cholesterol rich diet (28). 

The results of the DPPH assay have demonstrated that the free radical scavenging activity of oleuropein was more potent than that of synthetic antioxidant BHT (butylated hydroxyl toluene) (28). The results of laboratory tests and the DPPH, TEAC, FRAP and hydroxyl radical scavenging activity assays in our study confirmed that the antioxidant power of oleuropein increased with increasing its concentration. We also observed that the antioxidant capacity in serum and brain increased significantly in the oleuropein and diazepam receiving groups compared to the PTZ receiving group; and serum MDA levels in the diazepam receiving group decreased significantly compared to the PTZ receiving group, which indicates the increased antioxidant potency in serum and decreased lipid peroxidation due to epileptic seizures. The subsequent treatment with flumazenil caused a significant increase in lipid peroxidation and decrease in antioxidant capacity, which shows that flumazenil exacerbates nerve cell damage by inhibiting the anticonvulsant effects of oleuropein. Evidence on rodents has indicated that the seizure leads to production of high levels of inflammatory mediators in the brain regions (4). These inflammatory mediators are involved in the production and development of epileptic activities. Certain inflammatory cytokines such as IL-6, IL-1β, and TNF-α are upregulated in activated microglia and astrocytes, therefore a cascade of the inflammatory events is initiated in the neurons and endothelial cells in the blood brain barrier (BBB) (4, 29). The levels of IL-1 receptor in nerve cells increase dramatically immediately after the seizure. The binding of IL-1β to IL-1R1 stimulates SRC kinase, resulting in the phosphorylation of the NR2B subunit of the NMDA receptor, a key receptor of glutamate in the seizure process. As a result of this action, NMDA receptor-mediated Ca2+ influx into neuron cells is increased by IL-1β, and this effect plays role in increasing excitotoxicity and exacerbates seizure (30, 31). Anti-inflammatory compounds can therefore help to decrease the severity epileptic seizures. The anti-inflammatory property of oleuropein has been studied (13, 32). In a model of spinal cord damage, treatment with oleuropein (20mg/kg) immediately after spinal cord damage caused decrease in the expression of TNF-α, inducible nitric oxide synthase (iNOS), IL-1β, and COX (6). Consumption of olive oil has been found to significantly inhibit the activities of iNOS and inflammatory mediators after cerebral hypoxia-reoxygenation (33). Another study showed that daily consumption of olive oil in the rats with cerebral hypoxia-reoxygenation caused decrease in iNOS, lactate dehydrogenase activity, and lipid peroxidation in the damaged brain slides (9). Consumption of oleuropein (1 and 6 h) after trauma caused decrease in the expression of TNF-α, IL-1β, iNOS, nitrotyrosine and protein kinase A, neutrophil infiltration and the formation of poly-ADP-ribose (32). Our study showed that the IL-1β levels in the oleuropein group significantly decreased when compared to the PTZ group, but flumazenil treatment significantly increased IL-1β levels in the oleuropein receiving mice compared to the PTZ group.

Cognitive disorders are common among patients with epilepsy. In epilepsy, degen-eration of the nerve cells occurs in the limbic areas including CA_3_, CAL, and dentate gyrus in the hippocampus, amygdala and entorhinal cortex (34). Nerve cell damage in the hippocampus leads to memory and learning impairments. The role of the hippocampus in memory processes has been well explained, and damages to different regions of the brain can cause severe forgetfulness (34, 35). PTZ-induced epilepsy in rats leads to impaired passive avoidance memory and significantly reduces latency to enter the dark chamber in the shuttle box test (36). PTZ-induced epileptic seizure is associated with impairments in spatial memory, learning and passive avoidance memory in the laboratory animals (36). 

The current study showed that secondary latency in the shuttle box test significantly increased in the mice treated with oleuropein and PTZ; in other words, oleuropein could significantly improve the passive avoidance memory in the PTZ receiving mice and prevent memory loss through preventing damage to the tissues involved in memory and learning. 

## Conclusion

In this study, oleuropein treatment caused a significant increase in seizure latency and a significant decrease in the frequencies of the whole body seizures and frequent spinning and jumping in the shuttle box test, but treatment with flumazenil (GABA receptor antagonist) in the oleuropein receiving mice did not cause any significant change in anticonvulsant effects; and given no significant change in GLT-1 gene expression in the oleuropein group compared to the PTZ group, it is less likely for oleuropein to exert anticonvulsant effect through the GABAergic system. The results of different phyrochemical tests confirmed the potent antioxidant activity of oleuropein; and with respect to decreased IL-1β gene expression in the oleuropein group compared to the PTZ group, oleuropein antiepileptic property is due to its anti-inflammatory and antioxidant properties. Taken together, oleuropein can be recommended as an herbal AED as well as a drug for the learning and memory impairments related to epilepsy. 
